# Exploring the genetic and genomic connection underlying neurodegeneration with brain iron accumulation and the risk for Parkinson’s disease

**DOI:** 10.1038/s41531-023-00496-y

**Published:** 2023-04-06

**Authors:** Pilar Alvarez Jerez, Jose Luis Alcantud, Lucia de los Reyes-Ramírez, Anni Moore, Clara Ruz, Francisco Vives Montero, Noela Rodriguez-Losada, Prabhjyot Saini, Ziv Gan-Or, Chelsea X. Alvarado, Mary B. Makarious, Kimberley J. Billingsley, Cornelis Blauwendraat, Alastair J. Noyce, Andrew B. Singleton, Raquel Duran, Sara Bandres-Ciga

**Affiliations:** 1 Molecular Genetics Section, Laboratory of Neurogenetics, National Institute on Aging, National Institutes of Health, Bethesda, MD USA; 2 Center for Alzheimer’s and Related Dementias (CARD), National Institute on Aging and National Institute of Neurological Disorders and Stroke, National Institutes of Health, Bethesda, MD USA; 3 Department of Neurodegenerative Disease, UCL Queen Square Institute of Neurology, University College London, London, UK; 4 Institute of Neurosciences “Federico Olóriz”, Centro de Investigación Biomédica, Universidad de Granada, Granada, Spain; 5 Laboratory of Neuropharmacology. Dept. Medicine and Life Sciences, Universitat Pompeu Fabra, Barcelona, Spain; 6 Department Human Physiology, Faculty of Medicine, Biomedicine Research Institute of Malaga (IBIMA C07), University of Malaga, Malaga, Spain; 7 Montreal Neurological Institute, McGill University, Montréal, QC Canada; 8 Department of Human Genetics, McGill University, Montréal, QC Canada; 9 Department of Neurology and Neurosurgery, McGill University, Montréal, QC Canada; 10 Data Tecnica International, Washington, DC USA; 11 Department of Clinical and Movement Neurosciences, UCL Queen Square Institute of Neurology, London, UK; 12 Preventive Neurology Unit, Centre for Prevention, Detection and Diagnosis, Wolfson Institute of Population Health, Queen Mary University of London, London, UK

**Keywords:** Genetic association study, Genomics

## Abstract

Neurodegeneration with brain iron accumulation (NBIA) represents a group of neurodegenerative disorders characterized by abnormal iron accumulation in the brain. In Parkinson’s Disease (PD), iron accumulation is a cardinal feature of degenerating regions in the brain and seems to be a key player in mechanisms that precipitate cell death. The aim of this study was to explore the genetic and genomic connection between NBIA and PD. We screened for known and rare pathogenic mutations in autosomal dominant and recessive genes linked to NBIA in a total of 4481 PD cases and 10,253 controls from the Accelerating Medicines Partnership Parkinsons’ Disease Program and the UKBiobank. We examined whether a genetic burden of NBIA variants contributes to PD risk through single-gene, gene-set, and single-variant association analyses. In addition, we assessed publicly available expression quantitative trait loci (eQTL) data through Summary-based Mendelian Randomization and conducted transcriptomic analyses in blood of 1886 PD cases and 1285 controls. Out of 29 previously reported NBIA screened coding variants, four were associated with PD risk at a nominal *p* value < 0.05. No enrichment of heterozygous variants in NBIA-related genes risk was identified in PD cases versus controls. Burden analyses did not reveal a cumulative effect of rare NBIA genetic variation on PD risk. Transcriptomic analyses suggested that *DCAF17* is differentially expressed in blood from PD cases and controls. Due to low mutation occurrence in the datasets and lack of replication, our analyses suggest that NBIA and PD may be separate molecular entities.

## Introduction

Neurodegeneration with brain iron accumulation (NBIA) represents a group of inherited heterogeneous neurodegenerative disorders characterized by iron accumulation and the presence of axonal spheroids in the basal ganglia and other brain areas^1^. The worldwide prevalence of NBIA in the general population is estimated between 1–3/1,000,000 individuals, adding these disorders to the group of ultra-rare orphan diseases^2^. In view of the rarity of these disorders and its diverse clinical presentation, NBIA can often go unrecognized, underdiagnosed, or misdiagnosed.

In the brain, the substantia nigra, putamen, globus pallidus and caudate nucleus have the highest iron concentration, and total iron content increases with age^3^. Brain iron is crucial for important processes such as the synthesis of myelin and neurotransmitters and oxygen transport^3,4^. Higher nigral iron concentrations have been shown in Parkinson’s Disease (PD) patients in postmortem studies repeatedly^5,6^. This excess of intracellular iron is associated with oxidative stress, lipid peroxidation, cellular dysfunction, and neuronal death in PD and thus potentially playing a role in PD pathogenesis^7,8^.

To date, mutations in 13 genes have been associated with autosomal dominant (*FTL*), autosomal recessive (*ATP13A2, PANK2, PLA2G6, FA2H, CP, C19orf12, COASY, GTPBP2, DCAF17, VAC14*) and X-linked forms of NBIA (*WDR45, RAB39B*), and are involved in a wide range of molecular processes affecting mitochondrial function, coenzyme A metabolism, lipid metabolism and autophagy^9^. Typically, NBIA disorders present with variable and complex phenotypes including parkinsonism, dystonia, intellectual disability, and cognitive decline^1^. The NBIA clinical spectrum is broad and there is increased awareness of clinical overlap between different NBIA disorders as well as with other diseases such as Parkinson’s disease (PD). In PD, it has been widely suggested that genetic components contributing to disease might include rare genetic variants of small or moderate effect, where functional and deleterious alleles might exist^10^. Furthermore, it has been previously predicted that variability in genes causing young-onset autosomal recessive neurological diseases contribute to late-onset neurological diseases. In fact, homozygous and compound heterozygous loss-of-function mutations in *TREM2* have been previously associated with an autosomal recessive form of early-onset dementia, while when found in heterozygous state confer moderate risk for Alzheimer’s disease^11,12^. Similarly, homozygous and compound heterozygous mutations in *GBA* are responsible for Gaucher’s disease while heterozygous variants are a well-validated risk factor for PD^13^.

In PD, iron accumulation is a cardinal feature of degenerating regions in the brain and has been implicated in mechanisms that precipitate cell death^14^. Iron is a transition metal involved in several cellular functions such as neuronal metabolism, DNA synthesis, oxygen transport, mitochondrial respiration, and myelination in the brain^15^. Furthermore, it is implicated in production and turnover of some neurotransmitters such as dopamine, epinephrine or serotonin^16^. Iron tends to accumulate with age mainly in the cortex and the nuclei of the basal ganglia, including the globus pallidus, putamen, and caudate nucleus; and an excessive accumulation in these regions is associated with neurodegenerative disorders^17^. Evidence suggests abnormal iron levels in the brains of PD patients and a role for iron dysregulation in the disease^18^. Interestingly, NBIA-related genes are involved in mitochondrial function and autophagy, dysfunction of which is implicated in PD, suggesting there could be shared molecular pathways and gene networks between NBIA and PD^19^. Furthermore, autopsy examination of NBIA-genetically confirmed cases has demonstrated Lewy bodies in some subforms, linking the pathology of both diseases^20^.

The aim of the present study was to explore the relationship between genes known to contain mutations that cause autosomal dominant/recessive NBIA and PD etiology. Using the largest genetic and genomic datasets of PD cases and controls to date including the Accelerating Medicines Partnership Program for Parkinson’s Disease (AMP-PD) and the UKBiobank (UKB), we screened for the presence of known and rare pathogenic mutations linked to NBIA in PD. We further examined whether a genetic burden of variants in NBIA-linked genes could contribute to the risk of developing PD by performing single gene, gene-set, and single variant association analyses. Finally, to investigate the potential effect of changes in NBIA-linked genes expression in PD compared to healthy individuals, we assessed publicly available expression quantitative trait loci (eQTL) results from GTEx v8, BRAINEAC, and transcriptomics data from AMP-PD.

## Results

### Genetic screening of NBIA-related genes in whole-genome and whole-exome sequencing data of Parkinson’s disease cases and controls

#### ATP13A2

Genetic variants in the *ATPase Cation Transporting 13A2* (*ATP13A2*) gene, located on chromosome 1, have been previously associated with Kufor-Rakeb syndrome, spastic paraplegia type 78, and parkinsonism^21–23^. A total of 36 protein-coding variants were present in the screened datasets, of which four missense variants had a higher frequency in cases versus controls. A summary of variants with a higher frequency in cases can be found in Table 1 while additional variant information is listed in the supplemental. The variants found in AMP-PD (p.I946F, p.A249V, p.T12M) have been previously associated with PD in individuals of European ancestry although their pathogenicity is still unclear^24,25^. The p.T402M variant, found in UKB, has been previously described in a homozygous individual with ataxia-myoclonus syndrome with an age at onset of 38 and lacking parkinsonism^26,27^. The report postulates that together, progressive ataxia with action myoclonus along with lack of parkinsonism may suggest a new phenotype linked to *ATP13A2*. In our UKB cohort, the p.T402M heterozygous mutation carrier was a male PD case with age at recruitment of 55 years. This mutation was absent in controls.

**Table 1:** Summary of variants in NBIA related genes with higher frequency in PD cases versus controls, SNP = Single Nucleotide Polymorphism, *p* = *P*-value.

SNP	Gene	p (AMP-PD)	p (UKB)	Cohort
chr1:16988161:T:A	ATP13A2	0.147	–	AMP
**chr1:16996487:G:A**	**ATP13A2**	–	**0.024**	**UKB**
chr1:17000494:G:A	ATP13A2	0.502	–	AMP
chr1:17005754:G:A	ATP13A2	0.264	–	AMP
chr3:149183513:C:T	CP	0.067	0.887	AMP
chr3:149198448:T:A	CP	–	0.047	UKB
chr3:149199783:G:A	CP	0.648	0.260	AMP
chr3:149206202:T:A	CP	–	0.427	UKB
**chr16:74719154:G:A**	**FA2H**	–	**0.024**	**UKB**
chr16:74774524:C:T	FA2H	0.824	0.985	AMP
chr16:74774662:G:C	FA2H	0.875	–	AMP
chr19:29702747:T:C	C19orf12	–	0.609	UKB
chr19:29702977:C:A	C19orf12	0.875	–	AMP
chr19:29702977:C:G	C19orf12	0.264	–	AMP
**chr19:29708268:G:A**	**C19orf12**	–	**0.024**	**UKB**
chr19:48966341:G:T	FTL	0.875	–	AMP
chr20:3889237:A:T	PANK2	–	0.694	UKB
chr20:3907997:A:G	PANK2	0.114	–	AMP
chr20:3910812:A:G	PANK2	0.439	–	AMP
**chr20:3912486:T:A**	**PANK2**	–	**0.024**	**UKB**
chr20:3912490:D:3	PANK2	–	0.199	UKB
chr20:3918695:G:A	PANK2	0.439	0.106	AMP
chr22:38113560:C:T	PLA2G6	–	0.427	UKB
chr22:38126390:T:C	PLA2G6	0.875	0.069	AMP
chr22:38132881:C:T	PLA2G6	0.467	0.523	AMP
chr22:38132917:C:T	PLA2G6	0.264	–	AMP
chr22:38133007:G:A	PLA2G6	0.875	–	AMP
chr22:38143275:C:T	PLA2G6	0.875	–	AMP
chr22:38169326:G:A	PLA2G6	0.151	0.514	AMP

Bold entries indicate statistical significance at a nominal *p* value < 0.05.

#### CP

The *Ceruloplasmin (CP*) gene, located on chromosome 3, is closely related to iron metabolism in the brain^28^. Four out of 29 variants had a higher frequency in cases than controls. Notably, p.R793H and p.P477L, were seen in both AMP-PD and UKB. Reports have linked lower *CP* levels with PD development, and *CP* gene mutations have been associated with substantia nigra hyperechogenicity using transcranial sonography^29^. In addition, hyperechogenicity is a common finding in idiopathic PD patients and many studies suggest that it is an important risk marker of future PD^30,31^. One mutation originally associated with substantia nigra (SN) hyperechogenicity, although listed as likely benign, is p.R793H^32^. Our study identified the p.R793H variant in both homozygosity and heterozygosity with higher frequency in cases than controls both AMP-PD and UKB. However, it did not reach statistical significance for either dataset. Of note, we observed biallelic heterozygosity in one case and two controls from UKB carrying both the p.P477L and p.R793H mutations. The p.I392F variant was only present in UKB data. A three base pair deletion at this position has been associated with aceruloplasminemia, a disorder in which iron gradually accumulates in the brain and other organs^33^. In addition, this is a multi-allelic variant for which the risk allele identified in our analyses differs from those reported on HGMD.

#### FA2H

In the *Fatty Acid 2-Hydroxylase* (*FA2H*) gene, located on chromosome 16, we found 28 coding variants out of which two missense variants had a higher frequency in cases. The variant p.E78K appeared in both datasets and is associated with the fatty acid hydroxylase-associated neurodegeneration(FAHN)/hereditary spastic paraplegia (SPG35) phenotype although no conclusions were drawn about its significance^34^. The p.E78K mutation has also been reported in a patient with an age at onset of 10 with a positive family history of spastic paraplegia and was labeled as a putative pathogenic variant^35^. Our PD carriers with the p.E78K mutation had an age of inclusion of 70 and 52 for AMP-PD and 69 for UKB. The case/control frequency for p.E78K did not reach significance in our analysis. For the p.T207M mutation, all previously reported carriers were compound heterozygous with additional mutations in *FA2H* and had the spastic paraplegia phenotype^22,34,36^. In our study, we found one male UKB case with an age at inclusion of 65 carrying the p.T207M mutation. This mutation was absent in controls and was the only *FA2H* mutation that reached statistical significance.

#### C19orf12

Variation in the *Chromosome 19 open reading frame 12* (*C19orf12*) gene, located on chromosome 19, has been associated with the mitochondrial membrane protein-associated neurodegeneration (MPAN) subtype of NBIA which presents with cognitive decline progressing to dementia, prominent neuropsychiatric abnormalities, motor neuronopathy, and parkinsonism movement abnormalities^37,38^. Out of 18 coding variants present, four missense variants in our datasets showed a higher frequency in cases versus controls, with only one proving significant and previously linked to the MPAN phenotype in the literature. In AMP-PD data, we found two multiallelic variants, p.G65V present in one case and one control and p.G65A present in just one case and no controls. In two patients reported in the literature, compound heterozygosity for p.G65V and p.G69RfsX10 variants led to the MPAN phenotype^38^. In addition, another two patients were reported to have this phenotype; one carrying the compound heterozygous mutations p.G65V and p.P60L and the other carrying a homozygous p.G65V variant^39^. The p.K142E variant, present it our data, has been seen in compound heterozygosity in patients with the MPAN NBIA phenotype^40–42^. Lastly, we found the p.P60L mutation at statistical significance present in one UKB case and absent in controls. The carrier under study was a female PD patient with an age at inclusion of 66 and no other notable *C19orf12* mutations. The p.P60L variant has been previously detected in an MPAN patient who also carried the p.G65E *C19orf12* mutation^38^.

#### FTL

Our study identified only one missense variant (p.E104) out of nine with a higher frequency in cases than controls in the *Ferritin Light Chain* (*FTL*) gene, located on chromosome 19. The p.E104 mutation was only of note in the AMP-PD dataset and was present in one case and one control (F_A = 2.02 × 10^−4^, F_U = 1.62 × 10^−4^). The p.E104 variant has been reported as causing an L-ferritin deficiency phenotype^38,43^.

#### PANK2

Variation in the *Pantothenate Kinase 2* (*PANK2*) gene, located on chromosome 20, has been previously associated with pantothenate kinase-associated neurodegeneration (PKAN), the most common subtype of NBIA^44^. Clinical symptoms include dystonia, dysarthria, muscular rigidity, poor balance, and spasticity^44,45^. We found six out of 68 distinct coding variants in our dataset with a higher frequency in cases. The p.G521R mutation was the only one present in both AMP-PD and UKB. This variant results in an unstable and inactive PanK2 protein and has been reported as the most common variant in *PANK2*^46^. It is often found in a compound heterozygous state in individuals presenting with the PKAN phenotype^46,47^. In AMP-PD, two PD cases, one male and one female with ages at inclusion of 54 and 67 respectively, carried the p.G521R mutation in heterozygous state compared to one control (F_A = 4.05 × 10^−4^, F_U = 4.05 × 10^−4^). In UKB, three cases, all males with an average age at inclusion of 62 years old, presented with the p.G521R mutation in heterozygous state compared to five controls (F_A = 1.36 × 10^−3^, F_U = 4.43 × 10^−4^). None of the PD p.G521R carriers from either cohort contained this mutation in compound heterozygous state.

#### PLA2G6

In the *Phospholipase A2 Group VI* (*PLA2G6*) gene, located on chromosome 22, we found 100 variants of which seven missense variants had a higher frequency in cases versus controls. These variants have been previously associated with both infantile neuroaxonal dystrophy (INAD) and PD^48–52^. INAD is a severe progressive psychomotor disorder, which presents before the third year of life, characterized by the presence of axonal spheroids throughout the central and peripheral nervous system^52–54^. The variant p.M470V is the only variant out of three associated with the INAD phenotype that was present in both the AMP-PD and UKB cohorts^55^. In addition, p.A343T has been associated with PD and we identified 77 cases and 86 controls carrying this mutation in AMP-PD (F_A = 1.58 × 10^−2^, F_U = 1.41 × 10^−20^) and 29 cases and 130 controls in UKB data (F_A = 1.31 × 10^−2^, F_U = 1.15 × 10^−2^)^49,55^. Of note, one of the p.A343T AMP-PD case carriers was a female homozygous for the mutation with an age at inclusion of 65. In addition, a heterozygous carrier of p.A343T in the UKB was a PD case and additionally carried the p.S34L mutation, also associated with PD^49,55^. This patient had an age at inclusion of 60. However, no *PLA2G6* mutation with a higher frequency in cases reached statistical significance.

### Transcriptomic analyses

**Fig. 1:**
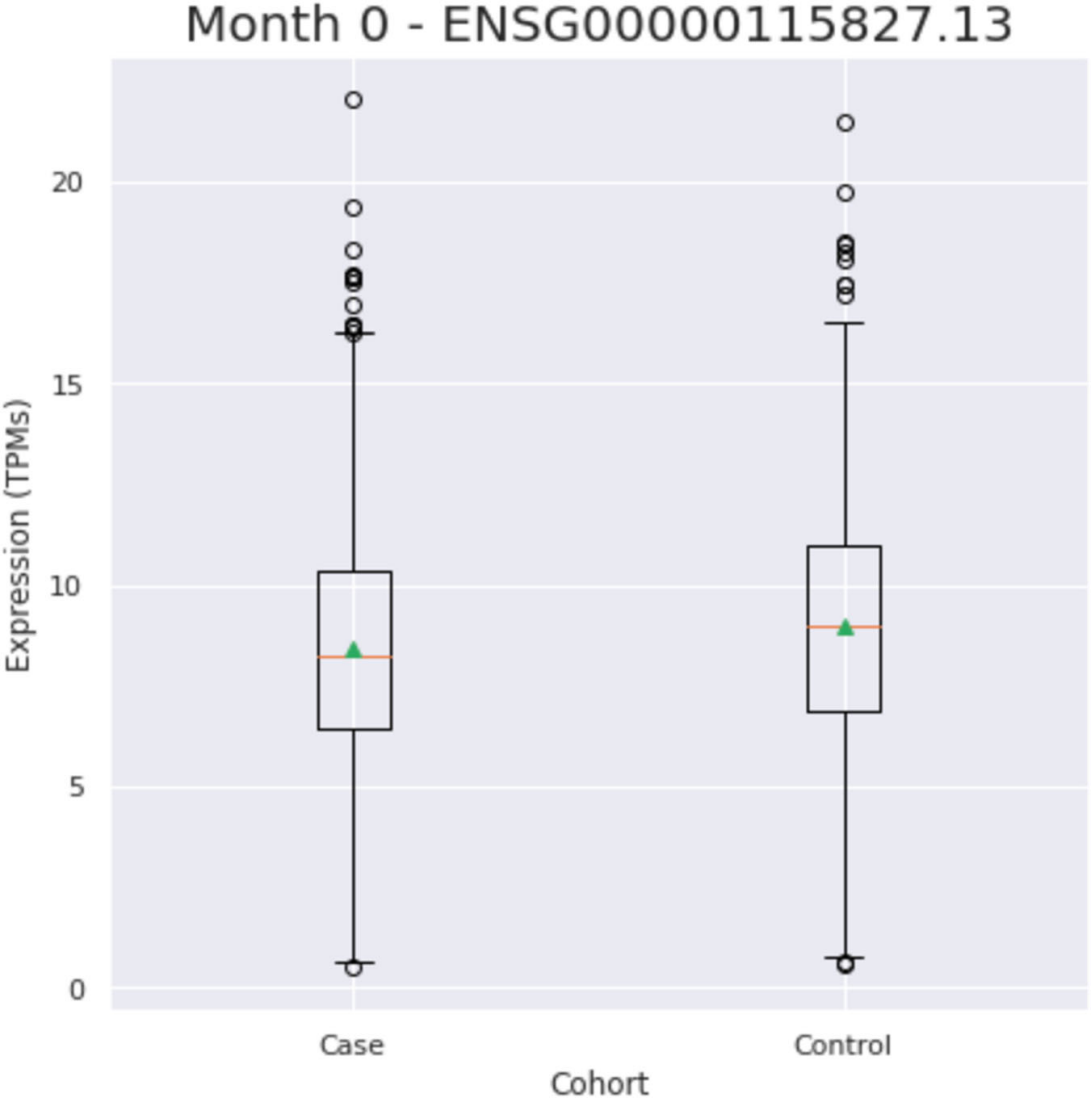
*DCAF17* expression in blood of PD cases versus controls. Center line indicates the median. The box bounds represent the first and third quartiles while the whiskers extend the box by 1.5× the interquartile range (IQR).

Two genes including *FA2H* (ENSG00000103089.8) and *CP* (ENSG00000047457.13) were removed for further analyses based on poor call rates (missingness rate > 0.33). Of the 10 NBIA genes left, *DCAF17* expression (ENSG00000115827.13) was found to be significantly different in cases versus controls (Fig. 1)(Table 2). To further investigate these results we looked into SMR analysis for the *DCAF17* gene. To prioritize SNV candidates, results were initially filtered by chromosome and base pair position for the *DCAF17* gene in hg19. Afterwards, the resulting candidates were filtered by the SMR multi p-value at a threshold of *p* < 0.05. SMR analyses for the *DCAF17* gene revealed four eQTL signals with a significant SMR *p*-value. Out of these four eQTL signals, only one was significant in both SMR analyses (*p* = 0.0024) and Nalls et al.^67^ GWAS (*p* = 0.0004). A summary of the DCAF17 SMR data can be found in the supplementary data.

**Table 2:** Results of transcriptomic analysis using blood PPMI data.

Gene	Ensemble ID	T-statistic	*P*-value
ATP13A2	ENSG00000159363.17	−0.5126	0.6083
**DCAF17**	**ENSG00000115827.13**	**−4.036**	**5.54E−05**
GTPBP2	ENSG00000172432.18	−0.3596	0.7192
VAC14	ENSG00000103043.14	−0.5266	0.5985
COASY	ENSG00000068120.14	0.4632	0.6432
C19orf12	ENSG00000131943.17	−1.446	0.1482
FTL	ENSG00000087086.14	0.655	0.5125
PANK2	ENSG00000125779.22	0.8218	0.4113

*DCAF17* is the only NBIA gene with significant differential expression in PD.

Bold entries indicate statistical significance at a nominal *p* value < 0.05.

### Cumulative effect of genetic variation through single gene and gene-set burden analyses

When considering the cumulative effect of rare variation on PD risk, single gene and gene-set burden analyses did not reveal any statistically significant differences between cases and controls at MAF < 0.01 or MAF < 0.03 in any of the three cohorts and after meta-analysis (Table 3).

**Table 3:** Results of burden analysis at two minor allele frequencies for NBIA genes in PD.

Gene	Variant Group	MAF	P_AMP	P_UKB	P_Meta	WeightedZ
*ATP13A2*	All	0.01	0.0904	0.8813	0.2811	0.6518
*ATP13A2*	All	0.03	0.6599	0.5547	0.7339	−0.6615
*ATP13A2*	Coding	0.01	0.9272	0.2949	0.6281	−1.0192
*ATP13A2*	Coding	0.03	0.6622	0.3033	0.5233	−1.2470
*C19orf12*	All	0.01	0.8789	0.5115	0.8089	1.6650
*C19orf12*	All	0.03	0.7091	0.5115	0.7305	1.2089
*C19orf12*	Coding	0.01	1.0000	0.4262	0.7897	1.6592
*C19orf12*	Coding	0.03	1.0000	0.4262	0.7897	1.6592
*COASY*	All	0.01	0.5435	0.0832	0.1853	−0.5970
*COASY*	All	0.03	0.5435	0.0832	0.1853	−0.5970
*COASY*	Coding	0.01	1.0000	0.0936	0.3154	0.4017
*COASY*	Coding	0.03	1.0000	0.0936	0.3154	0.4017
*CP*	All	0.01	0.3392	0.4920	0.4657	1.0089
*CP*	All	0.03	0.6747	0.4339	0.6524	0.2643
*CP*	Coding	0.01	0.6615	0.3514	0.5717	0.4431
*CP*	Coding	0.03	0.6615	0.2169	0.4221	0.1493
*DCAF17*	All	0.01	0.2382	0.2288	0.3721	−0.9574
*DCAF17*	All	0.03	0.2382	0.7715	0.8393	0.2144
*DCAF17*	Coding	0.01	0.1146	0.4555	0.6522	0.8371
*DCAF17*	Coding	0.03	0.1146	0.7161	0.8917	1.2486
*FA2H*	All	0.01	1.0000	0.3676	0.7355	−0.4057
*FA2H*	All	0.03	0.8598	0.3676	0.6801	−0.7388
*FA2H*	Coding	0.01	0.5816	0.5064	0.6546	−0.9324
*FA2H*	Coding	0.03	0.5397	0.5064	0.6278	−0.8240
*FTL*	All	0.01	0.4990	0.8460	0.7862	−0.1314
*FTL*	All	0.03	0.4990	0.8460	0.7862	−0.1314
*FTL*	Coding	0.01	0.5583	NA	NA	0.0555
*FTL*	Coding	0.03	0.5583	NA	NA	0.0555
*GTPBP2*	All	0.01	0.5471	0.1980	0.3491	−0.7209
*GTPBP2*	All	0.03	0.2766	0.4241	0.3687	−0.8013
*GTPBP2*	Coding	0.01	0.0696	0.3808	0.1227	−1.6865
*GTPBP2*	Coding	0.03	0.6000	0.3712	0.5572	0.7064
*PANK2*	All	0.01	0.3261	0.8209	0.6205	1.5979
*PANK2*	All	0.03	0.4622	0.9000	0.7809	0.8418
*PANK2*	Coding	0.01	0.7875	0.0507	0.1684	0.3729
*PANK2*	Coding	0.03	0.7875	0.0507	0.1684	0.3729
*PLA2G6*	All	0.01	0.7644	0.2124	0.4575	1.3405
*PLA2G6*	All	0.03	0.7661	0.3815	0.6518	0.4891
*PLA2G6*	Coding	0.01	0.9007	0.3588	0.6882	−0.9407
*PLA2G6*	Coding	0.03	0.8603	0.6690	0.8935	0.1710
*VAC14*	All	0.01	0.2814	0.4390	0.3819	−1.2446
*VAC14*	All	0.03	0.3986	1.0000	0.7653	−0.3362
*VAC14*	Coding	0.01	0.3683	0.7708	0.6414	0.0051
*VAC14*	Coding	0.03	0.3683	0.8155	0.6616	−0.0728

*MAF* Minor Allele Frequency, *P_AMP* *P* value in AMP_PD cohort, *P_UKB* *P* value in UKB cohort, *P_Meta* *P* value in meta analysis, *Weighted Z* Weighted Z Statistic.

## Discussion

Using the largest PD genetic and genomic datasets available to date, we conducted a comprehensive genetic assessment of NBIA related variants and genes for a potential role in PD etiology. We identified 29 previously reported NBIA coding mutations in a total of seven genes including *ATP13A2, CP, FA2H, C19orf12, FTL, PANK2,* and *PLA2G6* with a higher frequency in PD cases than controls. All phenotypes previously linked to the reported variants in NBIA genes involve neurological disorders, adding to the conclusion of clinical overlap between neurological diseases and the strong link between brain iron accumulation and a number of neurodegenerative disorders. However, only four out of the 29 variants of interest we reported had a statistically significant difference in frequency between cases and controls (*p* < 0.05), all only significant in UKB data. Within these four variants, one was found in *ATP13A2*, one in *FA2H*, one in *C19orf12*, and one in *PANK2*, always in only one case and no controls. Notably, none of these four variants have been previously associated with PD in the literature. Noting the very low mutation frequency in the current data and the lack of replication, we cannot conclude that the mutation spectrum contributing to NBIA phenotype overlaps with PD etiology. As such, despite enrichment for the risk allele in PD cases versus controls in four variants, these data suggest that in their heterozygous state NBIA variants are not frequently associated with risk of PD.

**Fig. 2:**
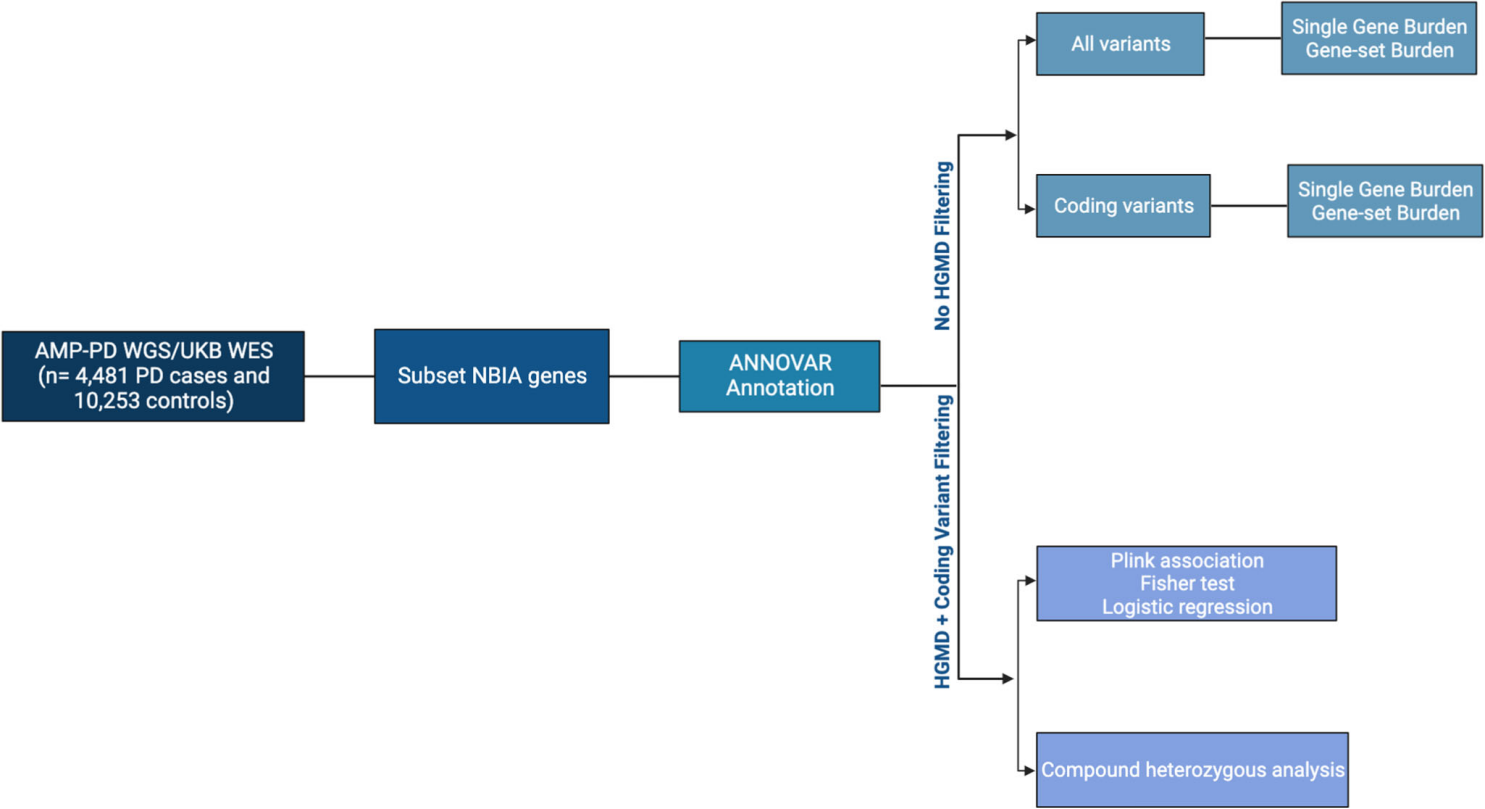
Overview of methods for association and burden analyses. Variants in NBIA genes were called in our PD and control cohorts and were then annotated and run through the analyses^69^.

We performed burden analyses at two different frequency levels, MAF < 0.01 and MAF < 0.03, on both individual genes and as a gene-set. Meta-analysis of SKAT-O results were not significant for any individual gene or as a gene-set at either frequency level, suggesting that a cumulative effect of rare variation on NBIA genes does not play a role on PD etiology (Fig. 2).

Transcriptomic analyses revealed that *DCAF17* is the only gene that is differentially expressed in blood from PD cases and controls. We further investigated this gene through Summary data-based Mendelian Randomization. One *DCAF17* eQTL signal was significant for both GWAS and SMR. The *DCAF17* gene is found on chromosome 2, encodes DDB1- and CUL4- associated factor 17, and is considered the cause of Woodhouse-Sakati syndrome, a rare neuroendocrine disorder that presents with neurological problems such as seizures and dystonia. While the exact role of the protein is still unclear, it is postulated that it may affect cellular activities such as growth, proliferation, and stress response specifically in the nucleolus of brain, liver, and skin cells^56^. It is possible that differing cellular stress responses between PD cases and controls may be leading to *DCAF17* differential expression. However, it is still unclear what is driving the significant *DCAF17* differential gene expression in our data and further assessments are needed.

This study has some limitations, mostly related to variant detection. Despite using the most well-powered PD datasets, it is nevertheless difficult to detect rare variants as evidenced by the number of variant counts of zero in both cases and controls. It is possible that other rare variants exist in NBIA genes that were not detected in our study. Therefore, we cannot fully elucidate the relationship between rare variants in NBIA genes and PD. In addition, due to a low number of non-European individuals found in the chosen datasets, we were underpowered to properly detect variation in other populations. Several the variants we identified have been reported in the literature in non-European populations, so a study encompassing a greater genetic diversity, with a larger number of non-European individuals, is warranted and would allow us to better characterize the relationship between NBIA and PD etiologies. In addition, as we have mainly focused on coding variants, it is also possible that non-detected variants, such as larger structural variant (SVs) events in NBIA genes, affect PD risk. There is increasing evidence that SVs are likely the main contribution to disease susceptibility and have a substantial effect in coding regions of the genome where they can result in deleterious alterations of the DNA sequence^57^. Therefore, further study into SVs would be an important step in identifying the relationship between NBIA and PD. Lastly, the cohorts currently available do not allow us to accurately perform a comprehensive clinical characterization of potential atypical symptoms nor are we able to perform clinicogenetic correlations exploring brain iron accumulation on MRI in PD cases with NBIA gene variants, therefore possibly drawing limited conclusions. In addition, as it has been previously suggested elevated nigral iron levels are unlikely to contribute to PD etiology but may vary with anti-parkinsonian drugs used for treatment^58^. Taking everything into account, we suggest that even though NBIA and PD share similar symptoms, they could be molecularly different entities supporting the notion that the mechanisms underpinning iron accumulation in PD are not shared with NBIA. Elevated nigral iron levels may not contribute to PD etiology and may vary with anti-parkinsonian drugs used for treatment or any other environmental factors.

## Methods

### Whole-genome sequencing data

Whole-genome sequencing (WGS) data was obtained from the Accelerating Medicines Partnership—Parkinson’s Disease Initiative release 2.5 (AMP-PD; www.amp-pd.org) which contained 3376 PD patients and 4610 healthy unrelated controls of European ancestry, with an average age at onset of 62 years in cases and age at collection of 72 years in controls. Ancestry was determined by principal component analysis versus the 1000Genomes populations and individuals deviating by more than six standard deviations from the European population mean were excluded from the analysis. Diagnosis criteria vary slightly between each subcohort within AMP-PD, but detailed cohort characteristics, as well as quality control procedures, are further described in https://amp-pd.org/whole-genome-data. Informed consent was obtained for all human participants in this cohort. Healthy unrelated controls include individuals with no neurodegenerative disease diagnosis nor any family history of such disease lacking any familial link to other individuals in our study. Data generation is described in detail by Iwaki and colleagues^59^. NBIA-related variants were extracted from the data by using PLINK1.9 and annotated with ANNOVAR^60,61^. Coding variants present in the Human Gene Mutation Database (HGMD) went through association analyses for risk of PD using Fisher’s exact test and logistic regression and adjusted by sex, age, and at least 5PCs to account for population substructure.

To assess the cumulative effect of multiple rare variants on the risk for PD, we performed single gene and gene-set burden analyses for all extracted variants under RVTESTS package v2.1.0 default parameters and adjusted by sex, age, and at least 5 PCs to account for population substructure^62^. Including all variants within the gene boundaries, a minimum allele count (MAC) threshold of 1 was applied. Sequence Kernel Association Test (SKAT-O) was performed considering two variant frequency levels: MAF < 1% and MAF < 3%. Age, sex, and at least 5 PCs, were accounted for as covariates.

### Whole-exome sequencing data

Whole-exome sequencing (WES) data was obtained from the UK Biobank 2021 release (UK Biobank; https://www.ukbiobank.ac.uk/), which contained 1105 PD patients and 5643 healthy unrelated controls of European ancestry, with an average age at recruitment of 63 years in cases and 64 years in controls. Ancestry determination follows that of AMP-PD. Disease diagnosis of individuals is determined by self-reports and hospital codes and healthy unrelated controls include individuals with no neurodegenerative disease diagnosis nor any family history of such disease lacking any familial link to other individuals in our study. Detailed cohort characteristics, as well as quality control procedures, are further described in https://www.ukbiobank.ac.uk/enable-your-research/about-our-data/genetic-data. Informed consent was obtained for all human participants in this cohort. Variant extraction, association tests, and burden tests follow the AMP-PD WGS pipeline.

### Transcriptomics data

Whole blood time progression gene expression data (gencode v29) from 0 to 24 months after first study visit was accessed from AMP-PD. We used PPMI (https://amp-pd.org/unified-cohorts/ppmi), PDBP (https://amp-pd.org/unified-cohorts/pdbp), and BioFIND (https://amp-pd.org/unified-cohorts/biofind) cohorts at the baseline of the study, 0 month time point (note BioFIND ‘Baseline’ is at ‘0.5’ month), including a total of 1886 cases and 1285 control samples. Expression data were quantified as transcripts per kilobase million (TPM) and quantile normalized before NBIA related genes values were extracted using Ensembl gene ids. Using the scikit-learn Python packages 19, residuals were calculated using linear regression, and the data were adjusted using age, sex, race, at least 5 PCs, cohort, and missingness rate as covariates. To test for significant differences in expression between the cases and controls, we performed a *t*-test with the residuals. We also used an additional dataset taken from the 24-month time point in the PDBP and PPMI cohorts of 841 cases and 386 controls to confirm our findings.

### Summary based Mendelian Randomization

SMR is a mendelian randomization (MR) method that uses summary-level data to test if an exposure variable (i.e. gene expression) and outcome (i.e., trait) are associated because of a shared causal variant. In order to distinguish pleiotropy from linkage, the heterogeneity in dependent instruments (HEIDI) method was applied to each tested single nucleotide variant (SNV)^63^. SMR and HEIDI analysis were conducted using the SMR package^64^.

In order to conduct the analysis, we used PD GWAS summary statistics as well as methylation and eQTL meta-analysis summary statistics^65,66^. PD GWAS summary statistics from Nalls et al.^67^ contained 17 datasets which consisted of 37,688 PD cases, 18,618 proxy cases, and 1,417,791 controls^67^. The methylation data we used consists of brain and blood gene expression data from a meta-analysis conducted by Qi et al.^65^. All eQTL analyses were performed to test for potential in cis association between methylation site and SNV. In order to achieve this, SNVs within 1 Mb of the probes of interest were selected^65^. The eQTL summary statistic data used were generated from peripheral blood and came from the meta-analysis conducted by Wu et al.^68^. All of the SNPS from Wu et al. are located within 2 Mb of a probe^68^.

In order to prioritize SNP candidates, results were initially filtered by chromosome and base pair position for the *DCAF17* gene in hg19. Afterwards, the resulting candidates were filtered by the SMR multi p-value at a threshold of *p* < 0.05.

### Reporting summary

Further information on research design is available in the Nature Research Reporting Summary linked to this article.

## Supplementary information

Supplementary Tables

Reporting Summary

## Data Availability

Data used for this publication is available through the AMP-PD and UKBiobank websites upon request. Data access is dependent on approval of a Data Usage Agreement. AMP-PD Main Website: www.amp-pd.org; AMP-PD Cohort Information: https://amp-pd.org/whole-genome-data; UKBiobank Main Website: https://www.ukbiobank.ac.uk/; UKBiobabank Cohort Information: https://www.ukbiobank.ac.uk/enable-your-research/about-our-data/genetic-data.

## Code availability

Code used in this study can be found at our GitHub page: https://github.com/neurogenetics/NBIA_PD.
