# Supplementary material for: Exploring the genetic and genomic connection underlying neurodegeneration with brain iron accumulation and the risk for Parkinson’s disease
Source: NPJ Parkinsons Dis. 2023 Apr 6;9:54. doi: 10.1038/s41531-023-00496-y (PMC10079978; doi:10.1038/s41531-023-00496-y)
Supplement: Supplementary file 1 — Supplementary Tables [file 41531_2023_496_MOESM1_ESM.docx]

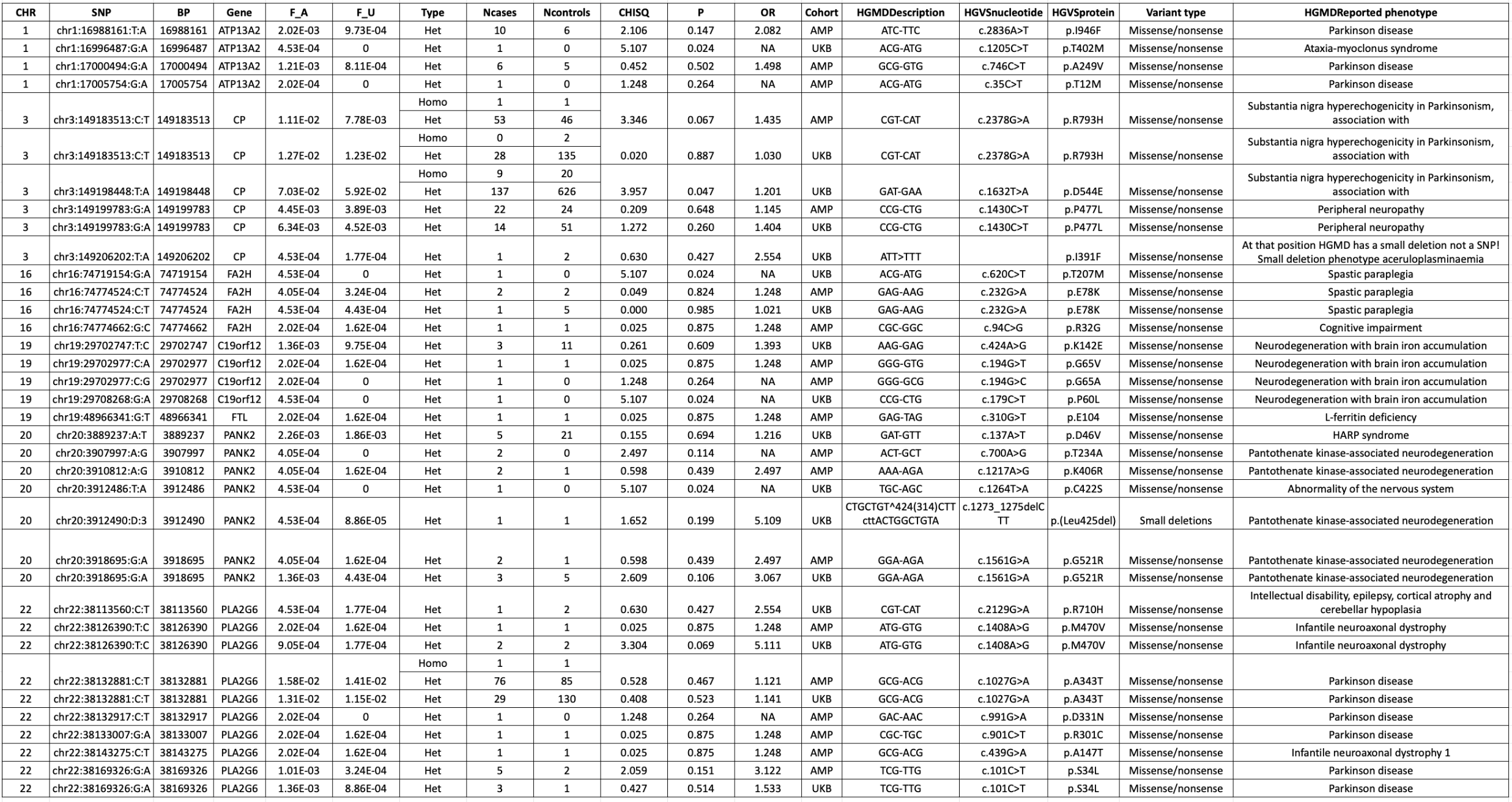


**Supplementary Table 1**: Screening of variants in NBIA related genes. CHR = Chromosome, SNP=Single Nucleotide Polymorphism, BP= Base Pair, F_A= Frequency Affected, F_U= Frequency Unaffected, Ncases= Number of cases, Ncontrols= Number of controls, CHISQ= Chi-squared, P= P value, OR= Odds Ratio


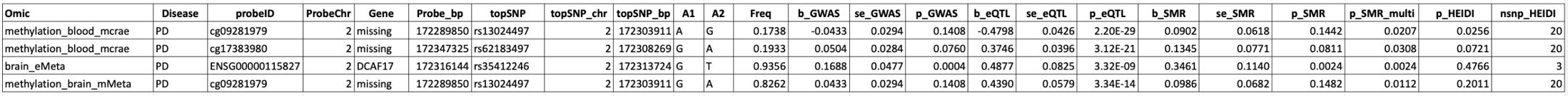


**Supplementary Table 2:** SMR results for *DCAF17*. Chr= Chromosome, BP= Base Pair, A1= Major Allele, A2= Minor Allele, b= Beta, se= Standard Error, p = p-value
