# Supplementary material for: Exploring the genetic and genomic connection underlying neurodegeneration with brain iron accumulation and the risk for Parkinson’s disease
Source: NPJ Parkinsons Dis. 2023 Apr 6;9:54. doi: 10.1038/s41531-023-00496-y (PMC10079978; doi:10.1038/s41531-023-00496-y)
Supplement: Supplementary file 2 — Reporting Summary [file 41531_2023_496_MOESM2_ESM.pdf]

nature portfolio

Corresponding author(s): Sara Bandres Ciga

Last updated by author(s): Mar 10, 2023

Reporting Summary

Nature Portfolio wishes to improve the reproducibility of the work that we publish. This form provides structure for consistency and transparency in reporting. For further information on Nature Portfolio policies, see our Editorial Policies and the Editorial Policy Checklist.

Statistics

For all statistical analyses, confirm that the following items are present in the figure legend, table legend, main text, or Methods section.

n/a	Confirmed	
☐	☒	The exact sample size (*n*) for each experimental group/condition, given as a discrete number and unit of measurement
☐	☒	A statement on whether measurements were taken from distinct samples or whether the same sample was measured repeatedly
☐	☒	The statistical test(s) used AND whether they are one- or two-sided<br>*Only common tests should be described solely by name; describe more complex techniques in the Methods section.*
☐	☒	A description of all covariates tested
☐	☒	A description of any assumptions or corrections, such as tests of normality and adjustment for multiple comparisons
☐	☒	A full description of the statistical parameters including central tendency (e.g. means) or other basic estimates (e.g. regression coefficient) AND variation (e.g. standard deviation) or associated estimates of uncertainty (e.g. confidence intervals)
☐	☒	For null hypothesis testing, the test statistic (e.g. *F*, *t*, *r*) with confidence intervals, effect sizes, degrees of freedom and *P* value noted<br>*Give P values as exact values whenever suitable.*
☒	☐	For Bayesian analysis, information on the choice of priors and Markov chain Monte Carlo settings
☒	☐	For hierarchical and complex designs, identification of the appropriate level for tests and full reporting of outcomes
☒	☐	Estimates of effect sizes (e.g. Cohen's *d*, Pearson's *r*), indicating how they were calculated

Our web collection on statistics for biologists contains articles on many of the points above.

Software and code

Policy information about availability of computer code

Data collection	No software used
Data analysis	Plink1.9, ANNOVAR, Human Gene Mutation Database, RVTESTSv2.1.0, SMR

For manuscripts utilizing custom algorithms or software that are central to the research but not yet described in published literature, software must be made available to editors and reviewers. We strongly encourage code deposition in a community repository (e.g. GitHub). See the Nature Portfolio guidelines for submitting code & software for further information.

Data

Policy information about availability of data

All manuscripts must include a data availability statement. This statement should provide the following information, where applicable:

- Accession codes, unique identifiers, or web links for publicly available datasets
- A description of any restrictions on data availability
- For clinical datasets or third party data, please ensure that the statement adheres to our policy

Data used for this publication is available through the AMP-PD and UKBiobank websites upon request. Data access is dependent on approval of a Data Usage Agreement.
AMP-PD Main Website: www.amp-pd.org
AMP-PD Cohort Information: https://amp-pd.org/whole-genome-data

UKBiobank Main Website: https://www.ukbiobank.ac.uk/
UKBiobabank Cohort Information:
https://www.ukbiobank.ac.uk/enable-your-research/about-our-data/genetic-data

Human research participants

Policy information about studies involving human research participants and Sex and Gender in Research.

Reporting on sex and gender	Publicly available data, sex was self reported for both AMP-PD and UKBiobank. No sex based analysis was performed, both included.
Population characteristics	Publicly available data, sex was self reported for both AMP-PD and UKBiobank. Only European ancestry used, based on principal component analysis. Disease diagnosis was obtained from self-reports and hospital codes. Informed consent was obtained from all participants in the study.
Recruitment	Self-enrollment for UKBiobank , AMP-PD harmonizes various cohorts
Ethics oversight	NA

Note that full information on the approval of the study protocol must also be provided in the manuscript.

Field-specific reporting

Please select the one below that is the best fit for your research. If you are not sure, read the appropriate sections before making your selection.

☒ Life sciences ☐ Behavioural & social sciences ☐ Ecological, evolutionary & environmental sciences

For a reference copy of the document with all sections, see nature.com/documents/nr-reporting-summary-flat.pdf

Life sciences study design

All studies must disclose on these points even when the disclosure is negative.

Sample size	4,481 PD patients and 10,253 healthy unrelated controls were included. These were the numbers available to us from UKBiobank and AMP-PD for our two testing groups.
Data exclusions	Related individuals were excluded to normalize for familial proxy cases. Non-european ancestry were excluded to account for genetic differences given ancestry.
Replication	No replication was performed as we used the largest PD datasets for our analysis.
Randomization	Experimental groups were determined by disease status. Sex, age, and 5PCs were taken as covariates.
Blinding	Blinding not relevant here, as we only took already publicly available data.

Reporting for specific materials, systems and methods

We require information from authors about some types of materials, experimental systems and methods used in many studies. Here, indicate whether each material, system or method listed is relevant to your study. If you are not sure if a list item applies to your research, read the appropriate section before selecting a response.

Materials & experimental systems

n/a	Involved in the study	
☒	☐	Antibodies
☒	☐	Eukaryotic cell lines
☒	☐	Palaeontology and archaeology
☒	☐	Animals and other organisms
☒	☐	Clinical data
☒	☐	Dual use research of concern

Methods

n/a	Involved in the study	
☒	☐	ChIP-seq
☒	☐	Flow cytometry
☒	☐	MRI-based neuroimaging
